# Miniplate for Osteosynthesis in a 9-Year-Old with Symphysis Fracture: Clinical Report

**DOI:** 10.5005/jp-journals-10005-1222

**Published:** 2013-10-14

**Authors:** Ila Srinivasan, Naveen Kumar, Udhya Jaganathan, Arihant Bhandari

**Affiliations:** Professor and Head, Department of Pedodontics and Preventive Dentistry, MR Ambedkar Dental College, Bengaluru, Karnataka India, e-mail: drilasri@yahoo.com; Senior Lecturer, Department of Pedodontics and Preventive Dentistry MR Ambedkar Dental College and Hospital, Bengaluru, Karnataka India; Postgraduate Student, Department of Pedodontics and Preventive Dentistry, MR Ambedkar Dental College and Hospital, Bengaluru Karnataka, India; Postgraduate Student, Department of Pedodontics and Preventive Dentistry, MR Ambedkar Dental College and Hospital, Bengaluru Karnataka, India

**Keywords:** Symphysis fracture, Open reduction and rigid internal fixation, Miniplate, Osteosynthesis

## Abstract

Osteosynthesis using minimum material in pediatric mandibular fractures is the key, due to the limited space available in the mandible, especially in the mental foramen and apical region. There is an important role of open reduction and rigid internal fixation in re-establishing facial height, width and projection. During the early years of growth and development, there is a high osteogenic potential of the bones. The thick periosteum allows for rapid consolidation and remodeling at the site of fracture. Primary teeth have short, bulbous crowns which compromise stable maxillomandibular fixation during fracture reduction and stabilization using traditional methods. Further, stability of the fractured segments may be hampered because of the displaced or mobile permanent anterior teeth in the mixed dentition along the line of fracture. This clinical report outlines the use of miniplate with monocortical screws in a 9-year-old boy with symphysis fracture.

**How to cite this article:** Srinivasan I, Kumar N, Jaganathan U, Bhandari A. Miniplate for Osteosynthesis in a 9-Year-Old with Symphysis Fracture: Clinical Report. Int J Clin Pediatr Dent 2013;6(3):213-216.

## INTRODUCTION

The incidence of facial bone injury is less among children. The occurance of pediatric mandibular fractures increases to 5 % at the ages of 6 years or older because of the decrease in relative size of the cranium.^1^ Protective social environment which is child friendly and parental supervision in the early years of life, mitigate the likelihood of serious injury. Posnick et al^1^ provided significant epidemiological data to indicate that the majority of pediatric facial fractures were found in males and the largest group of patients were in the age range of 6 to 12 years. The most common cause of trauma was motor-vehicle accidents followed by falls, sports injuries and interpersonal altercations. Among the 55% of mandibular fractures reported, condylar fractures are the most common, followed by symphyseal region, body and lastly the angle of the mandible. Although falls are common during these years, children are involved in play and in athletic activity with peers at school, and in their homes with siblings and friends. Fractures that are displaced may require a open reduction and fixation. Management is complicated by mixed dentition that is inherently dynamic and unstable in such age groups. Depending on the age of the patient, compliance and severity of the fracture, maxillomandibular fixation may also be needed to ensure a stable occlusion.^2^ Miniplate osteosynthesis was first introduced by Michelet in 1973^3^ and further modified by Champy in 1975.^3^

Where 2 mini plates were applied in interforaminal region.^4-6^ To avoid damaging dental roots, screws were fixed monocortically. The general rule in surgery, namely ‘as little alloplastic material as possible but as much as necessary’ should therefore be applied here as well pediatric mandibular fractures require thoughtful consideration in management to avoid further injury to the developing dentition and significant growth disturbance. With rapid healing and remodeling characteristics of growing mandible even significant alterations of occlusion, discrepancies and alignment are rapidly resolved; where these could be the indications for the rigid fixations.^7^ This clinical report discusses a treatment alternative of placement of transoral monocortical miniplate at the inferior border of the mandible for reduction of a symphyseal fracture in a 9-year-old male patient.

## CLINICAL CASE REPORT

**Fig. 1:**
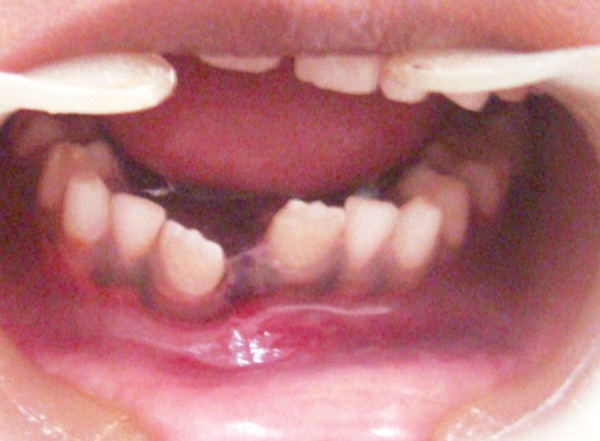
Preoperative photograph

A 9-year-old male patient presented to the Department of Pedodontics and Preventive Dentistry, with a chief complaint of pain on the lower jaw. The parents gave a history of fall from the gate while playing in the school. On external examination there was an obvious deviation of mandible to the right side. Intraoral examination revealed laceration of the soft tissue and an obvious fracture between permanent mandibular right and left central incisors (Fig. 1). The occlusion was also deranged and the child was in mixed dentition period. Patient was otherwise healthy, conscious, cooperative, well-oriented to time, place and person. There was no history of convulsions or vomiting.

On palpation, fractured fragments were mobile and tender. The teeth along the fractured segments were not mobile.

**Fig. 2:**
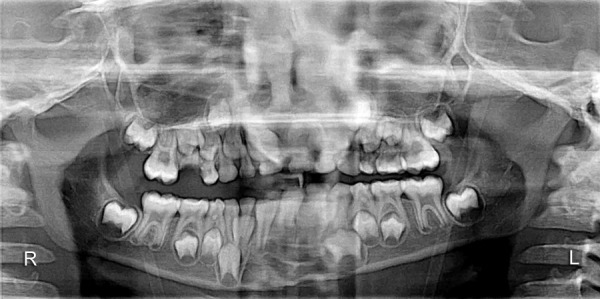
Preoperative radiograph

The orthopantomogram (Fig. 2) revealed radiolucent line extending from the superior border of symphysis between permanent mandibular right and left central incisors, to the base of the mandible. There was no overlap of the fractured fragments. Outer cortical boundary was irregular and had a step defect suggesting for an open reduction thus, diagnosing complete displaced symphysis fracture of the mandible. The presence of other concomitant fractures in the mandible however was ruled out.

**Fig. 3:**
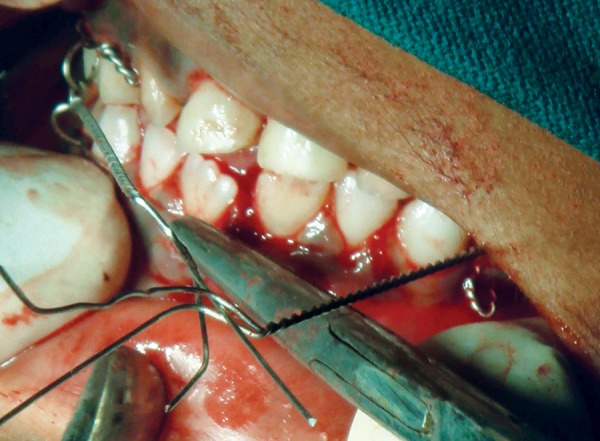
Eyelet wiring

**Fig. 4:**
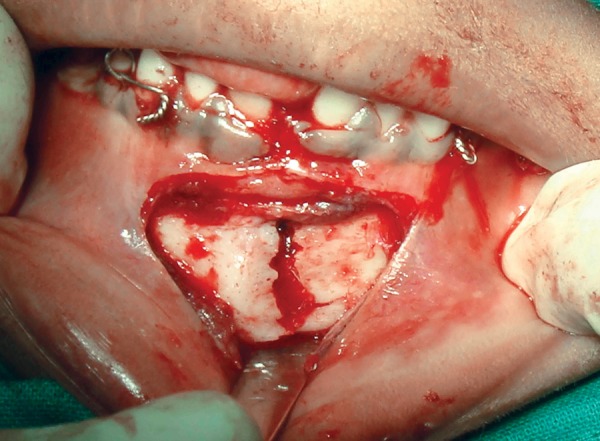
Digital approximation of fractured segments

The entire treatment plan was explained to the parents and the written informed consent was obtained.The child was admitted 1 day prior to the planned surgical procedure. Following the NPO guidelines, under general anesthesia, vestibular incision was made and the periosteum was exposed. Following eyelet wiring (Fig. 3) which was done for any need of doing an intermaxillary fixation later the fractured fragments were reduced by digital manipulation (Fig. 4). The monocortical plate was passively adapted along the contour of the external cortex without any gap between the plate and bone, at the inferior border of the mandible. Holes were drilled through the plate into the bone and two monocortical screws were fixed on either side of the fracture line to secure the fractured fragments together (Figs 5A and B).

Occlusion was rechecked and was found to be satisfactory as a result intermaxillary fixation was not done. Flap was repositioned and sutures were placed (Fig. 6). Patient was shifted to the postoperative ward and recovery from general anesthesia was uneventful. Patient was discharged at the end of second postoperative day with instructions of soft diet and maintenance of good oral hygiene. As the patient was prescribed antibiotics and analgesics prior to the surgical procedure same was continued 3 days after the procedure. The child was recalled after 1 week for a checkup and was followed-up every fortnightly till the plate was removed.

**Fig. 5A:**
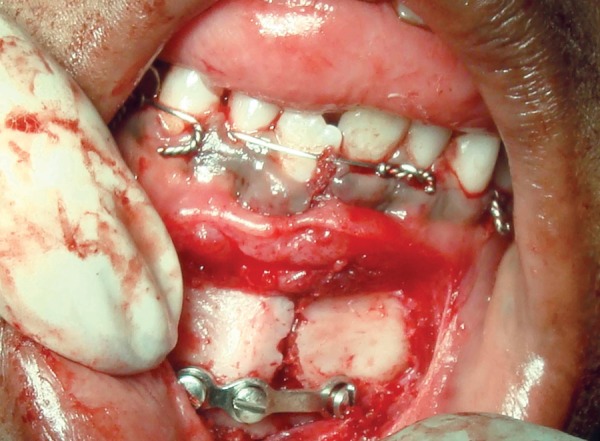
Miniplate and screw placement

**Fig. 5B:**
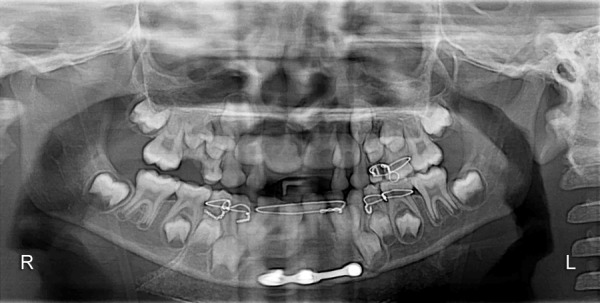
Immediate postoperative radiograph

**Fig. 6:**
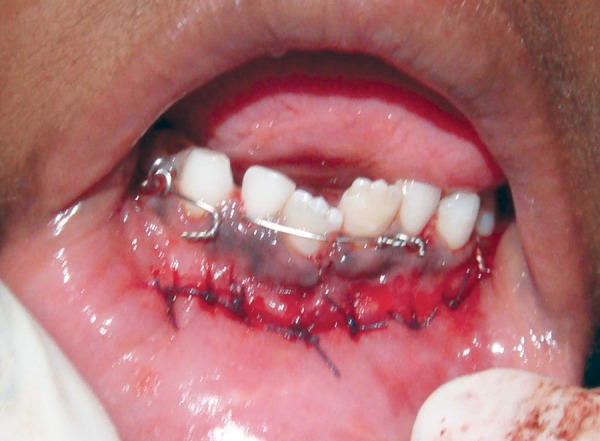
Sutures placed

**Fig. 7:**
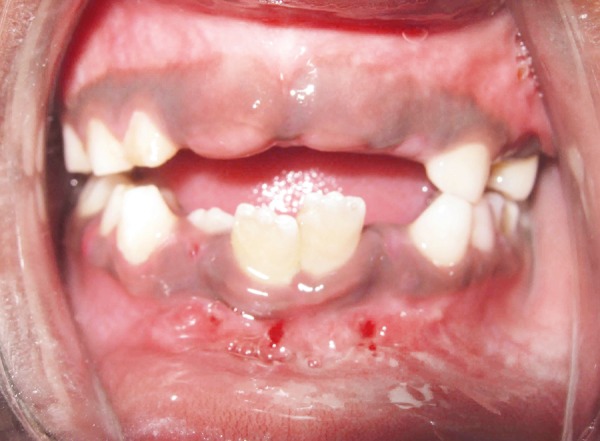
Three months postoperative photograph

**Fig. 8:**
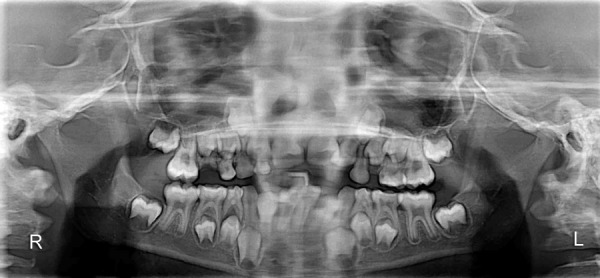
Three months postoperative radiograph

Plate removal was carried out at the end of 3 months. The consolidation of the fracture was confirmed clinically (Fig. 7) and radiographically (Fig. 8). Satisfactory healing and occlusion was observed.

## DISCUSSION

Fracture healing is a dynamic process in which masticatory forces are slowly intensifying and increasingly carried by the healing bone.^8^ Open reduction and internal fixation (ORIF) has become the standard of care for management of displaced fractures.^9^

This technique provides stable three-dimensional reconstruction, promotes primary bone healing, shortens treatment time eliminates the need for/permits early release of the intermaxillary fixation.^8^ The controversies of open reduction *vs* closed reduction of pediatric mandibular fractures remain. Facial fractures can be managed by either closed or open reduction. The type of fixation chosen depends on several factors: the age of the patient, the site of the fracture, the complexity of the injury and the approach that will be used to repair the fractures.^10-12^ Recent literature^1,13-15^ shows change in using open reduction and rigid internal fixation in fracture stabilization. The placement of miniplate and screw devices in mandibular fracture is probably only safe in the symphyseal and parasymphyseal regions at the lower border of the mandible after the eruption of the permanent incisors.^8^ The potential damage to tooth roots and follicle can be minimized with a careful technique where the plates can be placed only at the inferior border of the mandible using monocortical screws. The need for internal rigid fixation without intermaxillary fixation allows the child quicker resumption to a soft diet and also favors immediate jaw mobilization and an early return to dental hygiene habits.^16^ Open reduction when performed cautiously with minimal manipulation of the overlying soft tissues using an intraoral approach to reduce the risk of visible scarring is most beneficial. To prevent plate migration and the potential for interference with growth, early retrieval of any hardware is recommended in ages of patients less than 10 years.^17^ Nowadays resorbable fixation plates are used in addition to metallic mini- and micro- plates in the treatment of pediatric mandibular fractures.^12^ The reason for choosing metallic nonresorbable plate under absolute aseptic conditions as compared to a resorbable system in our clinical case, is because of the decreased stability that the resorbable plate provides in stabilizing the displaced segment. With the child being in the mixed dentition period the probable use of more than one resorbable plate may be required which can cause damage to the tooth roots as compared to a single metallic monocortical plate placed at the inferior border of the mandible. The resorbable systems as compared to metallic plates and screws have varying strengths which may resorb completely by a year.^18-20^ Any growth disturbance is controlled by early retrieval of internal rigid fixation systems. The use of monocortical plate with screws without the need for intermaxillary fixation was found to be efficient, well tolerated, economical, with ease of comfort for the patient in decreasing the immobilization time, decreased muscular atrophy and better oral hygiene maintenance. Since, the nutrition for the patient was not interfered with by the use of intermaxillary fixation, the healing was favorable. The plate was removed under mild sedation and under local anesthesia after a period of 3 months of placement and the healing was found to be satisfactory.

Fractures that are comminuted or displaced may require open reduction and internal fixation in restablishing facial height, width and projection. Metallic fixation plates are still used due to their predictive nature and ease of handling. The importance of placement of monocortical plate at the very inferior border of the mandible is crucial. Considerations must be given to early removal of internal fixation hardware once the union has been achieved. Parents should be counselled about the long-term follow-up of the child to observe any potential growth disturbances which may require additional treatment at a later date.
